# A Novel Model of Integrating Community Pharmacies With Telepharmacy Into the Telemedicine-Based Sick Child Care System in Japan

**DOI:** 10.7759/cureus.103738

**Published:** 2026-02-16

**Authors:** Shota Fujimura, Daisuke Matsubara, Yukifumi Monden, Yasushi Imai, Kazuhiko Kotani

**Affiliations:** 1 Department of Pharmacy, Jichi Medical University Hospital, Shimotsuke, JPN; 2 Division of Community and Family Medicine, Center for Community Medicine, Jichi Medical University, Shimotsuke, JPN; 3 Department of Pediatrics, Jichi Medical University, Shimotsuke, JPN; 4 Division of Clinical Pharmacology, Department of Pharmacology, Jichi Medical University, Shimotsuke, JPN

**Keywords:** community pharmacy, delivery system, sick child care, telemedicine, telepharmacy

## Abstract

Community pharmacies play a pivotal role in community healthcare. Recent technological advances, particularly the application of telemedicine in line with telepharmacy, are changing daily practice and creating new opportunities for pharmacies. We present a novel telemedicine-based sick child care system in Japan in which community pharmacies participate. In this system, when caregivers bring their child to a sick child care facility, on-site staff conduct teleconsultations with pediatricians. Childcare can be initiated immediately after the initial medical assessment. When medication is required, prescriptions are electronically transmitted to the family’s preferred community pharmacy. The pharmacy prepares and delivers medications directly to the childcare facility, where they are administered by on-site staff. Pharmacists can also provide teleconsultation as a type of telepharmacy to caregivers and on-site staff. Using this system, community pharmacies could contribute to the reduction in the time from medical assessment to the initiation of sick child care. This system also provides safe and improved care efficiency by sharing medication-related healthcare information with all staff involved. We present a novel approach with the possible involvement of community pharmacies in supporting sick child care domains that have not yet been widely introduced in the field. This system highlights the importance of close collaboration between pediatricians, childcare staff, and community pharmacists in digitally enabled community healthcare.

## Introduction

Community pharmacies and their services are essential for community healthcare. As highly trained healthcare professionals, pharmacists not only dispense and provide pharmaceuticals but also contribute to the improvement of healthcare in communities through a wide range of services, including preventive services (such as vaccination and screening for diseases) and patient education (for hypertension, diabetes, and obesity) [1,2]. Recently, community pharmacies have also been recognized as community hubs for residents because they are easily accessible and can connect with residents while collecting health-related information [3].

Pharmacy services have been provided through face-to-face communication and paper-based procedures [1-4]. However, the rapid advances in information and technology, accelerated by the COVID-19 pandemic, are influencing the pharmaceutical healthcare systems worldwide [2,4]. Telepharmacy, analogous to telemedicine, is a concept that refers to online-based provision of pharmaceutical services [5]. The National Association of Boards of Pharmacy defines “telepharmacy” as “the provision of pharmaceutical care through the use of telecommunications and information technologies to patients at a distance” [5]. Telepharmacy typically provides online services including medication order review, dispensing and compounding, drug information services, patient counseling, and therapeutic drug monitoring [4,6]. The application of telepharmacy, in combination with telemedicine, is changing daily practice, which leads to a paradigm shift in pharmacy practice and creates new opportunities for community pharmacies [4,6].

Telemedicine and telepharmacy have recently been adopted separately or in combination, showing efficacy and safety in primary care settings for both urgent and chronic care [7,8]. Telemedicine and/or telepharmacy have been adapted in certain facilities, such as nursing facilities for aged residents, and demonstrated improvements in patient outcomes and reductions of care-related costs [9].

However, there are no published models that explicitly integrate telemedicine, telepharmacy, and sick child care into a unified system. Here, we present a novel telemedicine-based sick child care system in Japan in which community pharmacies actively participate. By improving access to timely medical assessment and medical delivery, this system has the potential to address some of the structural and operational challenges within the sick child care system in Japan.

## Technical report

**Figure 1:**
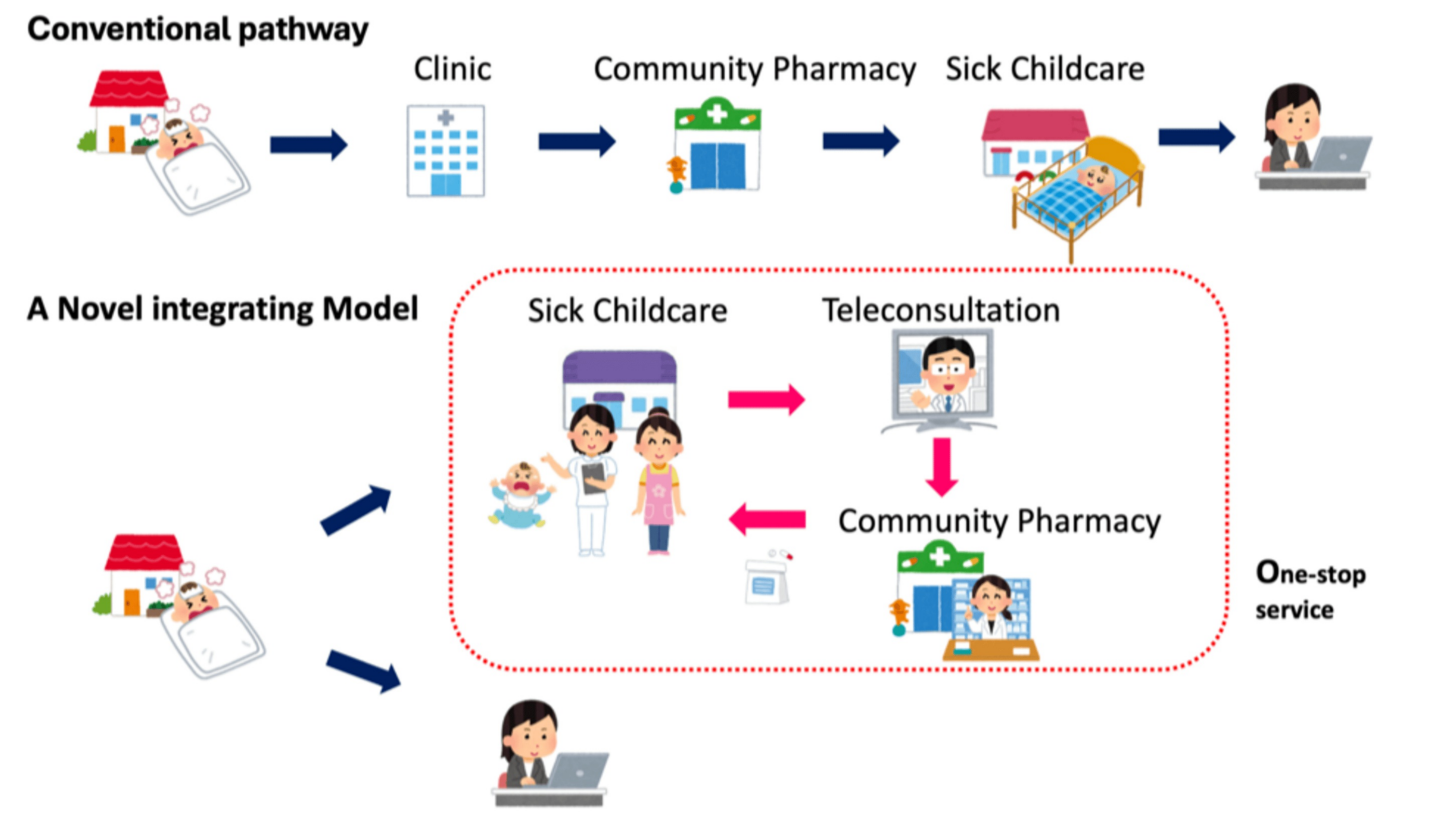
Images of one-stop service for sick child care at a single facility in combination with telemedicine and community pharmacy. Upper images: Conventional pathway for the initiation of sick child care in Japan, generally requiring multiple steps: visiting a clinic to see doctors for medical assessment → receiving a prescription → traveling to a pharmacy → obtaining the medication → finally bringing the child to the childcare facility. Each of these steps involves travel and waiting time, placing a substantial burden on the caregivers and children. Lower images: A novel integrating model. Caregivers directly bring their child to a designated sick child care facility. On-site staff (nurses) conduct remote consultations with pediatricians (Doctor-to-Patient With Nurse model). Rapid testing can be performed by on-site staff under the physicians’ instructions when needed. If permitted, sick child care can begin soon after the initial medical assessment, allowing caregivers to attend their workplace. When medication is required, prescriptions are transmitted electronically to the family’s preferred community pharmacy, where medication-related healthcare records are available. The pharmacy prepares and delivers the medications directly to the childcare facility, where they are administered by on-site staff. Pharmacists can also provide teleconsultations, as a type of telepharmacy, to the caregivers and on-site staff. Medication-related healthcare information through these steps was shared electronically with the staff involved.

Sick child care is a form of nursing care provided temporarily when parents cannot care for their child at home [10,11]. Generally, sick child care can be applied to children with mild acute illness or during recovery and requires an in-person physician’s assessment before initiation. Conventionally, in Japan, initiating sick child care requires a certain amount of time, often several hours, because caregivers must take multiple steps: visiting a clinic to see doctors for medical assessment with or without rapid testing for infection, receiving a prescription, traveling to a pharmacy, obtaining the medication, and finally bringing the child to the childcare facility. Each of these steps involves travel and waiting times, placing a significant burden on caregivers and children (Figure 1, upper images). Especially during epidemics of infectious diseases, the first step, i.e., visiting a clinic, can be difficult for caregivers because securing an appointment is often challenging.

In the novel system, at the initiation of sick child care, caregivers directly bring their child to a designated sick child care facility (rather than to a clinic), where telemedicine is conducted with an outpatient pediatrician in another facility for medical assessment, with the help of on-site staff (nurses) in a Doctor-to-Patient With Nurse model (Figure 1, lower Images) [12]. The telemedicine device used in our system provided video-based consultations and enabled the recording of clinical data, including vital signs, heart and lung sounds, and images of the throat, eardrum, and skin [12]. Rapid testing for infection can be performed by on-site staff following the physicians’ instructions when required. If permitted, childcare can begin soon after the initial medical assessment, allowing caregivers to attend their workplace. When a medication is required, prescriptions are transmitted electronically to the family’s preferred community pharmacy, where medication-related healthcare records are available. The community pharmacy prepares and delivers medications directly to the childcare facility, where they are administered by on-site staff. Pharmacists can also provide teleconsultation as a type of telepharmacy to caregivers and on-site staff. Medication-related healthcare information was shared electronically with the staff members involved.

Our previous pilot feasibility study showed that the time for childcare initiation was reduced by approximately 30 minutes in the integrated model group (involving two patients, a four-year-old boy with acute upper respiratory tract infection, and a three-year-old girl with acute pharyngitis) compared with the comparator group (using the conventional in-person physical assessment model, involving 26 episodes of 11 children, with a median age of 2.0 years, and an interquartile range of 1.0-3.8) [12]. No adverse events or serious complications, including delivery errors, occurred, suggesting the possible safety of this integrated model [12].

## Discussion

Our integration of community pharmacies into a telemedicine-based sick child care system has shown a possible reduction in the time of initiation of sick child care, possibly contributing to a decrease in caregivers’ and children’s burden. In addition, community pharmacies can contribute to safe and improved care by providing accurate information to facility staff and securing medications. Telepharmacy implementations often include distant dispensing and informed delivery of medications, offering flexible delivery pathways, including patient homes, healthcare centers, nursing homes, and community pharmacies [9,13]. However, our system is the first to explicitly integrate community pharmacies, telemedicine, and sick child care into a unified system. This system highlights the importance of close collaboration between pediatricians, childcare staff, and community pharmacists in digitally enabled community healthcare. Moreover, the system represents a novel approach that may inform future international practices and the possible involvement of community pharmacies in supporting sick child care domains that have not yet been widely introduced in the field.

Sick child care is a form of nursing care provided temporarily by nursery staff and nurses when their caregivers are unable to care for them. It is one of the government-supported child-rearing measures aimed at supporting the balance between work and family life. Such services are particularly important in the context of changing family demographics in Japan, including an increase in nuclear families, dual-income households, and single-parent households. In practice, sick child care is utilized predominantly by single mothers [10,11]. Young children with infectious diseases and mental and physical discomfort require individualized care considering their safety, symptoms, and development. However, regional studies have reported that a proportion of caregivers (approximately 30%) were unable to access sick child care services even when needed [10]. On some occasions, caregivers had to leave their children at home alone when sick child care was not available due to the limited number and insufficient capacity of sick child care facilities [10]. Therefore, there is a great demand for sick child care to ensure total care for sick children, support caregivers in child-rearing, and provide caregivers with work support. On the other hand, a national survey conducted by the Japanese Ministry of Health, Labour and Welfare reported a low utility rate of sick child care (45% in the acute phase and 16% during the recovery phase), suggesting unfamiliarity with sick child care in certain populations or regions and potential barriers to its usage [14].

One potentially major barrier to sick child care usage is the complex steps for initiation. As previously described, sick child care initiation requires a long time and multiple steps, which can place a burden on caregivers and limit the usage of sick child care. Considering the child-centered perspective, the rapid initiation of sick child care would be compassionate for sick children with mental and physical discomfort [10,11]. The integrated system could provide a solution via one-stop services for sick child care at a single facility, in combination with telemedicine and community pharmacies. This system could also provide safe and efficient care by sharing electronic medication-related healthcare information with all staff involved.

Beyond improving operational safety and efficiency, this system redefines the role of community pharmacies as integral community hubs in the era of rapid technological advancements [3]. By maintaining longitudinal medication records and engaging in continuous communication with both families and childcare staff, including physicians and nurses, community pharmacists can contribute to safer and more individualized childcare, including dose verification and formulation adjustments. This function is particularly important in telemedicine-based systems, where clinical decisions are made remotely and require robust medication management to ensure efficacy and safety, which can be supported by community pharmacists. Community pharmacies can provide accurate information to childcare staff and secure medications, thus contributing to the improvement of care. Such role redefining enhances the sustainability of sick childcare services, especially in regions facing a limited workforce. The easy access of this integrated system may maintain equities on working caregivers by reducing burdens, even in medically underserved areas, by leveraging online services [4-6].

Finally, this system aligns well with national digital health and community care strategies (the Doctor-to-Patient With Nurse model) [15] and may also be scalable to other settings, such as home-based care, acute childcare as school health services, and chronic disease management. These possibilities suggest that community pharmacies could play a key role in future digitally supported care systems. As such models expand, accumulating practical experience with telepharmacy and interprofessional collaboration may further support the integration of community pharmacies into these emerging care frameworks [16].

## Conclusions

We present a novel approach that integrates community pharmacies into a telemedicine-based sick child care system, a model that has not yet been widely implemented in this field. Our feasibility study showed the possible reduction of the time required to initiate sick child care services while maintaining safe operations. This system has the potential to reduce the burden on both caregivers and children, and may offer a practical solution to the complexity of conventional sick child care systems in Japan. Additionally, it can expand the role of community pharmacies from traditional dispensing functions to active participation in digitally enabled community healthcare. Owing to its remote and technology-driven design, the model is scalable and may be applicable to other regions worldwide, as well as to care settings beyond sick child care. Further investigation in larger patient populations is warranted to confirm the effectiveness and generalizability of this system.
